# The impact of multidisciplinary approaches on the outcomes of olfactory neuroblastoma treated with postoperative radiotherapy

**DOI:** 10.1002/cam4.6943

**Published:** 2024-03-18

**Authors:** Yuki Tsutsumi, Kazuhiro Omura, Yoshikazu Kijima, Masao Kobayashi, Nei Fukasawa, Teppei Takeda, Teru Ebihara, Satoshi Aoki, Nobuyoshi Otori, Hiromi Kojima, Manabu Aoki

**Affiliations:** ^1^ Department of Radiation Oncology Jikei University School of Medicine Tokyo Japan; ^2^ Department of Otorhinolaryngology Jikei University School of Medicine Tokyo Japan; ^3^ Department of Pathology Jikei University School of Medicine Tokyo Japan; ^4^ Department of Otorhinolaryngology Dokkyo Medical University Saitama Medical Center Saitama Japan

**Keywords:** Hyams grading, Kadish staging, multidisciplinary approach, olfactory neuroblastoma, recurrence, survival rates

## Abstract

**Background:**

We investigated the outcomes of postoperative radiation therapy for olfactory neuroblastoma (ONB) and our cross‐departmental collaboration to enhance the effectiveness of cancer treatment.

**Methods:**

We retrospectively evaluated 22 patients with ONB who underwent postoperative radiotherapy after tumor resection. En bloc resection was performed; pathology specimens were prepared in coronal sections; and irradiation fields were determined after discussion with radiation oncologists, head and neck surgeons, and pathologists.

**Results:**

The overall survival and local control rates were 95.5% and 100%, respectively, at a median 37‐month follow‐up. The 3‐ and 5‐year disease‐free survival (DFS) rates were 64.4% and 56.3%, respectively. Of the 22 patients, 9 (8 Kadish C and 1 Kadish B) had disease recurrence. Of the nine patients, five had positive margins and two had closed margins; cervical lymph node recurrence occurred in six, and distant metastasis with or without cervical lymph node recurrence occurred in three. DFS analysis of risk factors showed no statistically significant differences, but positive margins were a significant recurrence factor in multivariate analysis.

**Conclusions:**

The local control rate of ONB treated with postoperative radiation therapy was 100%. This may be attributed to cross‐departmental cooperation between head and neck surgeons, pathologists, and radiation oncologists, which resulted in accurate matching of CT images for treatment planning with the location of the tumor and positive margins. Longer follow‐up periods are required to evaluate the effectiveness of our strategy.

## 1 INTRODUCTION

Olfactory neuroblastoma (ONB), also referred to as esthesioneuroblastoma, is a rare malignant tumor that accounts for 2% of all nasal sinus tumors, with an incidence of 0.4 per million population per year.^1^ Although the tumor generally develops relatively slowly, there are some cases with relatively rapid progression.^2^ Depending on the grade and stage of the tumor, the 5‐year survival rate for low‐grade tumors is generally 80%, whereas that for high‐grade tumors is 40%.^1^ The distribution between the sexes was relatively equal, with a slightly higher incidence in males.^2^, ^3^ A bimodal age distribution for tumor occurrence has been reported, with a small peak observed in patients aged 10–20 years and a large peak in those aged 40–60 years.^3^, ^4^ ONB is a highly vascularized tumor that is homogeneously contrasted on gadolinium contrast‐enhanced magnetic resonance imaging (MRI).^5^ There are several staging systems, of which the Kadish and Hyams classifications are widely used. Kadish staging^6^, ^7^ is based on imaging findings, whereas Hyams grading^8^, ^9^ evaluates malignancy based on pathological features, such as the degree of mitosis or necrosis. Cervical lymph node metastasis (20%–30%)^10^, ^11^ and distant metastasis (approximately 10%) have been reported, regardless of the grade of the tumor.^1^, ^2^

**FIGURE 1:**
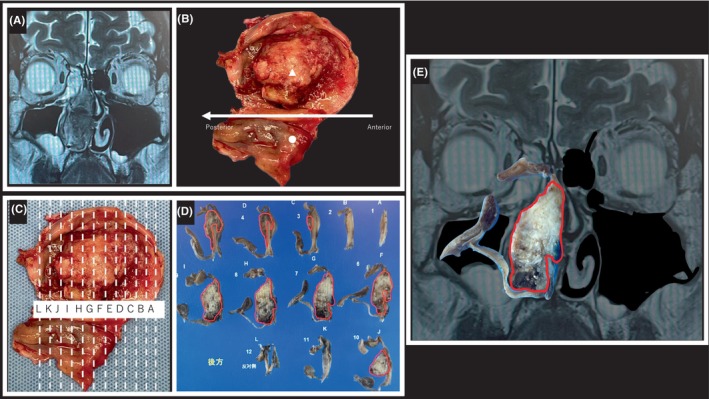
An example of surgical specimen and CT image of olfactory neuroblastoma. Preoperative coronal CT image (a) and the tumor resected by en bloc (b). The pathologist and head and neck surgeon worked together to make permanent surgical specimens by coronal section (c). The pathologist marked the permanent specimen tumor area in red to clarify the resection margin in the permanent specimen (d). The head and neck surgeon and radiation oncologist used the permanent specimen and CT images to contour the tumor beds and positive‐margin areas during radiotherapy planning (e).

Owing to the rarity of this tumor, there are no established treatment guidelines, and treatment options vary widely. Recent single‐center studies on definitive radiotherapy with particle beams such as protons and heavy ions have been reported.^12^, ^13^, ^14^, ^15^ The general treatment is surgical tumor resection, often followed by postoperative radiation therapy in patients at risk of recurrence.^16^, ^17^ Skull base reconstruction surgery is often associated with complications such as spinal fluid leakage and infection. However, skull base surgery using a 4‐layer reconstruction method was performed in our hospital without postoperative complications, such as spinal fluid leakage,^18^ which allowed for quick access to additional postoperative radiation therapy. All patients, except those with a low‐grade Kadish–Hyams classification and complete resection, were treated with additional postoperative radiation therapy. Pathological information such as resection margins is important for radiotherapy planning. Radiotherapy planning was performed using preoperative images, surgical records, and pathological reports; however, it was difficult to accurately determine surgical information. Therefore, we attempted to maximize the effectiveness of radiotherapy through cross‐departmental collaboration: Otorhinolaryngology, Pathology, and Radiation Oncology. First, the head and neck surgeon performed en bloc resection of the tumor while evaluating the tumor margins using intraoperative cytology (Figure 1A,B) and made permanent surgical specimens by coronal section in collaboration with the pathologist (Figure 1C). Second, the pathologist marked the permanent specimen tumor area in red to clarify the resection margin in the permanent specimen (Figure 1D). Finally, the head and neck surgeon and radiation oncologist used the permanent specimen and computed tomography (CT) images to contour the tumor beds and positive‐margin areas during radiotherapy planning (Figure 1E). These efforts have made it possible not only for head and neck surgeons but also for pathologists and radiation oncologists to visually match intraoperative information with pathology specimens and CT images of the nasal sinuses, which are organs with a complex anatomical structure, for use in pathological diagnosis and treatment planning. However, the benefits of prophylactic cervical lymph node irradiation for ONB remain controversial.^19^, ^20^, ^21^ Here, we administered radiation therapy only to the local area without prophylactic cervical lymph node irradiation, followed up the patients frequently, and performed cervical dissection immediately if recurrence was suspected.

In this study, we investigated the efforts of the Otorhinolaryngology, Pathology, and Radiation Oncology departments at our hospital to maximize the effectiveness of cancer treatment in ONB cases treated with postoperative radiation therapy. In addition, we reviewed the treatment outcomes, recurrence patterns, and optimal radiation fields for ONBs treated with postoperative radiotherapy.

## 2 MATERIALS AND METHODS

### 2.1 Data collection

This retrospective study was approved by the Ethics Committee of Jikei University Hospital on October 11, 2021, and May 12, 2023 (registration numbers 33‐257(10875) and 35‐009(11630)). Although the Ethics Committee waived the requirement for informed consent, efforts were made to directly notify patients about the conduct of this study. In addition, an opt‐out procedure was implemented whereby documents approved by the Ethics Committee were posted on the website so that patients could easily know about the research and be guaranteed the opportunity to reject the inclusion of their data in the study. Of 31 patients with ONB who underwent surgery at our otorhinolaryngology department between November 2014 and December 2020, 22 who underwent postoperative radiotherapy were retrospectively reviewed. All patients were evaluated using contrast‐enhanced CT and MRI and staged according to the Kadish staging system before surgery. Other information, such as age, medical history, surgical procedure, and pathology results, including the Hyams grading and margin status, were also reviewed. Significant factors were entered into multivariate analysis using the Cox proportional hazards model. Statistical analyses were performed using StatFlex V6.0 (Artech Co., Ltd, Osaka, Japan). *p*‐values <0.05 were considered to indicate statistical significance.

### 2.2 Treatment

Surgical procedures for ONB at our institution included en bloc resection of the tumor using extensive or endoscopic skull base tumor resection with a 4‐layer skull base reconstruction. The pathologist then prepared pathological specimens in the coronal section using a previously described method. Patients with Kadish stage A or B and Hyams Grade 1 or 2 with complete resection were followed up without radiotherapy, whereas all other patients underwent postoperative radiotherapy.

In this study, patients were treated with three‐dimensional conformal radiation therapy (3D‐CRT) or intensity‐modulated radiation therapy (IMRT).

Regarding the irradiation field, only the local area, including the surgical bed, was defined as the planning target volume (PTV) without prophylactic cervical lymph node irradiation in all cases. The PTV was determined through discussion with the head and neck surgeons and radiation oncologists based on the use of permanent specimens and CT images. The optic nerve, optic chiasm, retina, eyeball, lens, temporal lobe, and inner ear were set up as organs at risk. The basic prescribed dose was 54–60 Gy for the surgical bed and 66 Gy for areas with positive margins or where residual disease was suspected based on intraoperative findings. We did not administer chemotherapy in all cases.

### 2.3 Analysis

The overall survival (OS) and disease‐free survival (DFS) rates according to prognostic factors, such as the Kadish and Hyams classifications, were determined using the Kaplan–Meier method and compared using the log‐rank test for recurrent risk factors.

### 2.4 Follow‐up

In the first postoperative year, basic follow‐up clinical examinations with nasal fiber and neck palpation were conducted every month, and CT scans were taken every 3 months. In the second year, clinical examinations were conducted every 2 months, and CT scans every 4 months. From the third year, clinical examinations were conducted every 3 months, and CT scans every 6 months. MRI and positron emission tomography‐CT were performed if recurrence was suspected based on these tests.

The follow‐up period was from the start of postoperative radiotherapy to the last visit. OS was defined as the period from the start of postoperative radiotherapy to death from any cause or the last date of contact with a surviving patient. DFS was defined as the period from the start of postoperative radiotherapy to recurrence detected by imaging or nasal biopsy.

## 3 RESULTS

**FIGURE 2:**
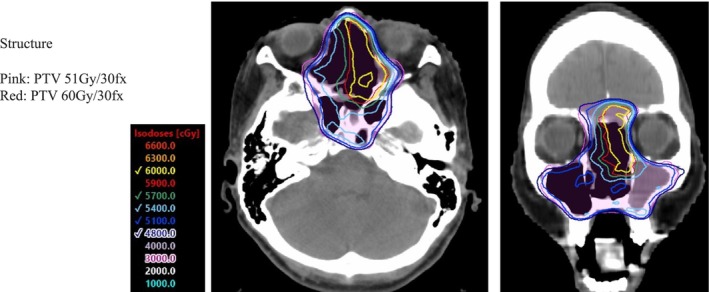
The isodose curves for the patient treated by intensity‐modulated radiation therapy (IMRT).

The study included 22 patients. Table 1 shows the patients' characteristics. The median patient age was 52 years (range, 29–76 years). Of them, 59% (13 cases) were male, and 41% (9 cases) were female. The most common chief complaint was epistaxis (54.5%), followed by nasal congestion (31.8%), and olfactory disorders (18.2%). The distribution according to the Kadish stage was A (9%), B (18.2%), and C (72.7%). There were no patients with Kadish stage D disease with distant or lymph node metastases. 3D‐CRT and IMRT were used in 9.1% and 90.9% of patients, respectively. The median total irradiation dose was 60 Gy (range, 50–70 Gy) (Figure 2).

**TABLE 1:** Patient's characteristics.

Variables	No. of patients (%) *n* = 22
Mean (range)	52 (29–76)
Age	
20–39	3 (13.6%)
40–59	12 (54.5%)
60–79	7 (31.8%)
Gender	
Male	13 (59%)
Female	9 (41%)
Presenting symptoms	
Epistaxis	12 (54.5%)
Nasal congestion	7 (31.8%)
Olfactory disorders	4 (18.2%)
Surgery	
Extensive skull base surgery	15 (68.2%)
Endoscopic endonasal skull base surgery	7 (31.8%)
Kadish staging	
Stage A	2 (9.0%)
Stage B	4 (18.2%)
Stage C	16 (72.7%)
Stage D	0 (0%)
Hyams grading	
Grade I	2 (9.0%)
Grade II	8 (36.4%)
Grade III	10 (45.5%)
Grade IV	2 (9.0%)
Margins	
Negative	10 (45.5%)
Closed	4 (18.2%)
Positive	8 (36.4%)

**FIGURE 3:**
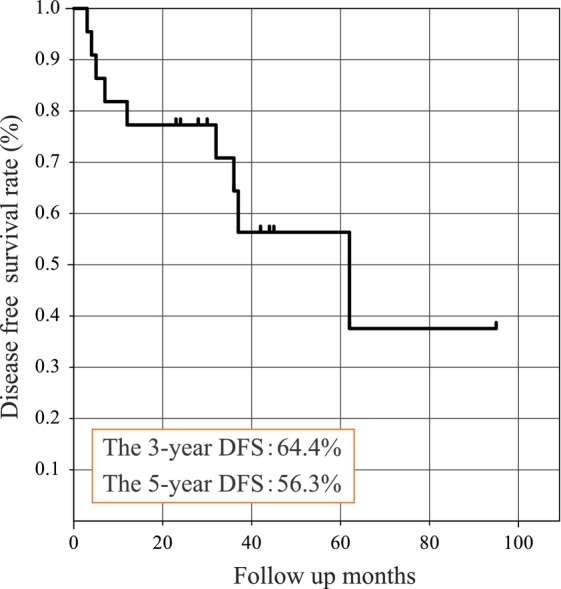
Disease free survival rate.

The median follow‐up period was 37 months (range, 5–95 months), and the local control rate was 100%, indicating no recurrence in the irradiation field. The 3‐ and 5‐year DFS rates were 64.4% and 56.3%, respectively (Figure 3). Nine of the 22 patients experienced disease recurrence. The median DFS period was 23.5 months (range, 3–95 months), and the median time to recurrence was 12 months (range, 3–62 months). Four of the nine patients showed recurrence after more than 2 years of follow‐up.

**FIGURE 4:**
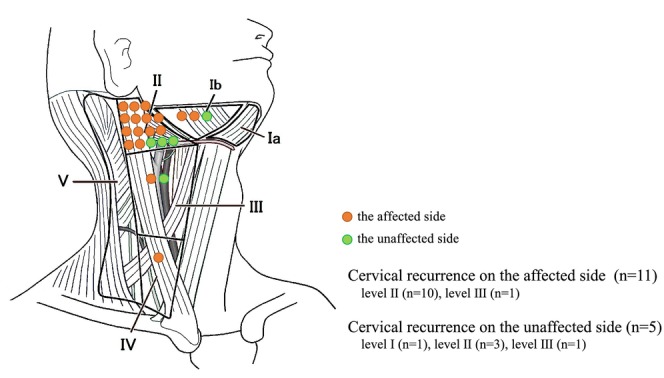
Site of recurrence of cervical lymph nodes.

The characteristics of the recurrent cases are presented in Table 2. All recurrent cases were Kadish C, except one Kadish B. Five of the nine recurrent cases had positive margins, and two had recurrence close to the margin. Three of the nine patients with distant metastasis with or without cervical lymph node metastasis at the time of recurrence diagnosis were treated with palliative radiation therapy for bone metastasis, CyberKnife for brain metastasis, and immunotherapy. Six of the nine patients with only cervical lymph node metastasis at the time of diagnosis of recurrence underwent immediate cervical lymph node dissection and postoperative radiation therapy if an *extranodal extension* was found. Three patients had no recurrence, and three patients subsequently developed distant metastases. In approximately one of the three cases in which distant metastasis developed after cervical lymph node dissection, metastasis was observed within the maxillary sinus on the affected side, followed by intracranial dissemination. In the other two cases, one had mediastinal lymph node and pulmonary metastases, and the other had cervical lymph node metastases on the unaffected side and dural metastases. The most common site of cervical lymph node recurrence was level II on the affected side; however, it was also observed at levels IB and III on the unaffected side (Figure 4).

**TABLE 2:** The characteristics of the recurrent cases.

Locations of recurrence	*n* = 9(%)	Case	Kadish	Hyams	Margins	DFS (month)
Cervical lymph node recurrence only	6 (66.7%)	1	Kadish C	Hyams III	Positive	3
		2	Kadish C	Hyams III	Positive	7
		3	Kadish C	Hyams II	Negative	12
		4	Kadish B	Hyams III	Negative	32
		5	Kadish C	Hyams III	Closed	36
		6	Kadish C	Hyams I	Positive	37
Cervical lymph node recurrence and distant metastases	1 (11.1%)	7	Kadish C	Hyams I	Positive	62
Distant metastases only	2 (22.2%)	8	Kadish C	Hyams III	Closed	4
		9	Kadish C	Hyams IV	Positive	5

**FIGURE 5:**
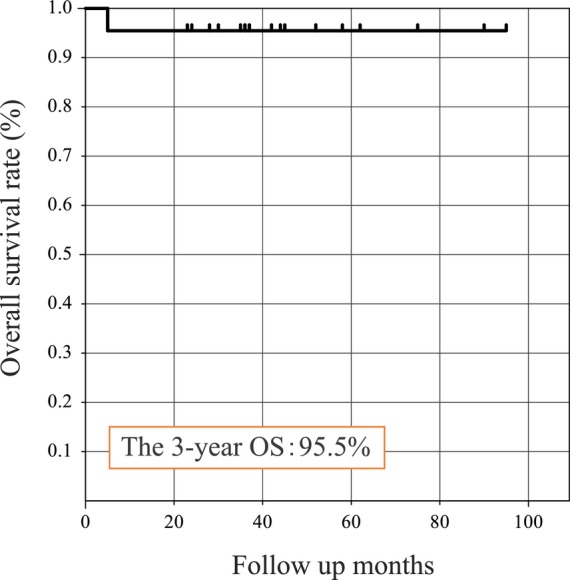
Overall survival rate.

The 3‐year OS rate was 95.5% (Figure 5). Only one patient died of ONB during the follow‐up period. The patient was a woman in her twenties and had Kadish C, Hyams Grade IV, and positive margins after surgery. She had brain, meningeal, and liver metastases at 4 months and died at 5 months of follow‐up.

**FIGURE 6:**
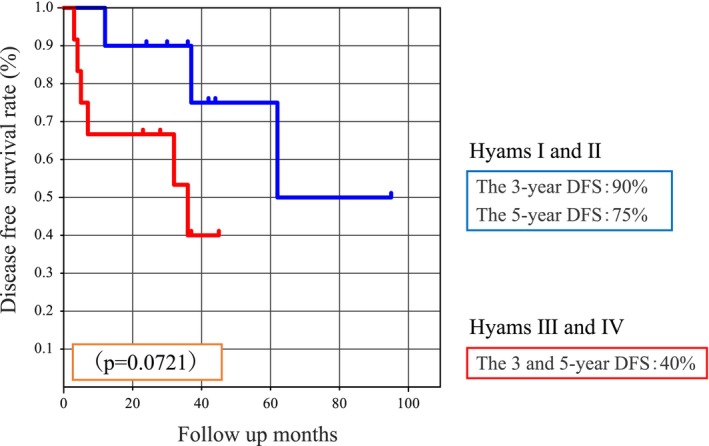
Disease‐free survival using the Hyams grading system.

**FIGURE 7:**
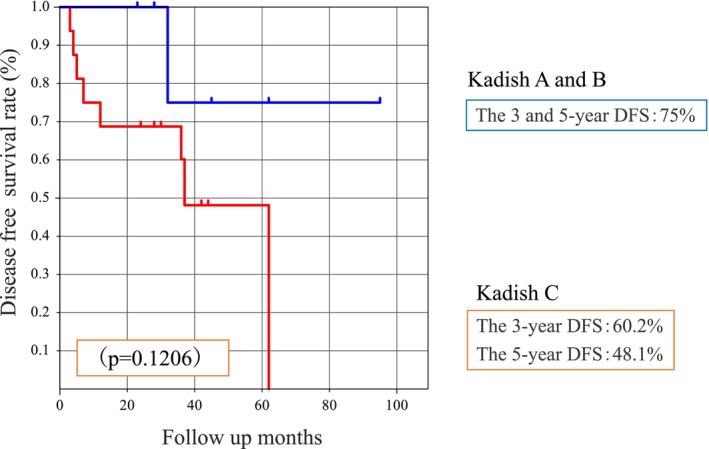
Disease‐free survival using the Kadish staging system.

DFS analysis using the Hyams grading system showed that the 3‐ and 5‐year DFS rates were 90% and 75% in the low Hyams (I and II) and 40% and 40% in the high Hyams (III and IV) groups, respectively, showing a tendency toward a worse prognosis in the high Hyams grading. However, these differences were not statistically significant (*p* = 0.0721) (Figure 6). Analysis using the Kadish staging system showed that the 3‐ and 5‐year DFS rates for patients in Kadish stages A and B were 75%, 60.2%, and 48.1%, respectively (*p* = 0.1206) (Figure 7). The 3‐ and 5‐year DFS rates for patients with negative or close margins were 64.3%, 62.5%, and 46.9%, respectively (*p* = 0.2930). However, the differences were not statistically significant. Patients with high‐grade Hyams (III and IV) and Kadish high‐stage (C) tumors tended to experience recurrence relatively early. Other factors, such as sex, total irradiation dose, and age, also showed no significant differences. Although the univariate analysis in our study showed no significant differences, we performed a multivariate analysis based on four common risk factors. Positive margin was the only significant prognostic factor (*p* = 0.0436) (Table 3).

**TABLE 3:** The multivariate analysis.

Variable	β	SE(β)	z score	*p*	RR	95% CI
Kadish	1.65646	1.20552	1.37406	0.1694	5.24072	0.49346–55.6584
Hyams	2.07913	1.09950	1.89099	0.0586	7.99752	0.92696–68.9999
Margin	1.81912	0.90141	2.01808	0.0436	6.16642	1.05378–36.0841
Age	1.26419	0.89161	1.41788	0.1562	3.54023	0.61673–20.3221

*Note*: AIC = 41.06369.

No serious acute adverse events occurred. Grade 2 pituitary dysfunction as a late adverse event was observed in one patient; it was suspected to be due to postoperative radiation therapy.

## 4 DISCUSSION

The Kadish staging classification based on imaging findings is a popular staging system for ONB. Kadish stage A is limited to the nasal cavity; stage B extends from the nasal cavity into the paranasal sinuses; stage C extends beyond the nasal and paranasal sinuses into the cranium; and stage D involves distant metastasis. Kadish C cases are most common (45%–61%) at the time of initial diagnosis because of delayed detection due to nonspecific nasal symptoms.^2^, ^7^, ^17^ Kadish A cases account for 6%–14%, and Kadish D cases with lymph node metastasis or distant metastasis account for 8%–12%.^2^, ^7^

Multivariate analysis showed that Kadish staging is an independent prognostic factor^22^; therefore, it is widely used in clinical settings. In addition to Kadish staging, several studies have shown that lymph node metastasis, intraorbital invasion, positive margins, and Hyams grading classification are associated with prognosis.^17^, ^21^ In this study, there was a tendency toward a worse prognosis in the high Hyams grade or Kadish stage and positive margin groups; however, the difference was not statistically significant. A positive margin was the only significant prognostic factor in multivariate analysis (*p* = 0.0436). The small number of patients enrolled in this study,^22^ despite being the largest sample size in a single institution in Japan, might have contributed to this lack of statistically significant differences. Therefore, future studies with longer follow‐up periods and larger sample sizes are warranted to investigate these differences.

Regarding the prognosis of ONB, previous studies have reported 5‐year survival rates of 72%–93% for Kadish A, 59%–88% for Kadish B, and 47%–52% for Kadish C.^1^, ^6^, ^23^, ^24^ Recurrence of ONB has been reported in approximately 30% of patients (range, 15%–70%), usually within 2 years of initial treatment.^1^ Although the median follow‐up in our study was not long (37 months; range, 5–95 months), the local control rate was 100%, and the 3‐year OS rate was 95.5% after postoperative radiation therapy, which compares favorably with previous studies, although the majority of cases were of Kadish C (72.7%). This good local control rate may be because the head and neck surgeons removed the tumors using en bloc rather than piecemeal resection, and the pathologists made pathological specimens in the coronal section, which enabled accurate matching of the planning CT images with the location of the tumor at the time of radiation therapy planning.

However, it is also known that recurrence can occur nearly 5 years after treatment due to the extremely slow‐growing nature of the tumor. Diaz et al. reported a single‐center study of ONB with long‐term follow‐up in 9 of 30 patients who developed recurrence, with a median time to recurrence of 4.67 years.^25^ Another single‐center retrospective study by Abdelmeguid et al. reported recurrence in 55 of 139 patients (39.6%), with a median time to recurrence of 3.5 years.^2^ In fact, the median time to recurrence at our hospital was 12 months, and five of the nine patients developed recurrence within 2 years after postoperative radiotherapy. However, four of nine patients experienced recurrence more than 2 years after postoperative radiotherapy, including a patient with Hyams Grade I, Kadish Stage C, and positive margins treated with 3D‐CRT who experienced cervical lymph node recurrence, dural dissemination, and subcutaneous metastasis 62 months after radiotherapy. The median follow‐up period of our study was 37 months, which was not significantly long, and further follow‐up is necessary.

Owing to its rarity, there is no established treatment strategy for ONB. Surgery alone may be chosen in patients with Kadish stage A and low Hyams Grade (I or II); however, some studies have reported that this may be associated with an increased risk of local recurrence, and careful patient selection should be made.^26^ The benefit of adding radiation therapy after surgical resection has been reported in many studies.^16^, ^17^ A meta‐analysis of 390 cases from 26 studies on ONB by Dulguerov et al. showed that a combination of surgery and radiation therapy was the optimal treatment^17^; the 5‐year survival rates were 65% for surgery with radiation therapy, 51% for chemoradiation therapy, 48% for surgery alone, 47% for surgery with chemoradiation therapy, and 37% for radiation therapy alone. For the prescribed dose of postoperative irradiation, a significant improvement in the 5‐year survival rate was reported for patients who received 54 Gy compared to those who received less than 54 Gy.^23^

There are also increasing reports on particle beam therapy as a definitive treatment in single centers. Particle beams, such as proton beams and heavy ions, are generally expected to reduce side effects because the Bragg peak, a characteristic of particle beams, allows the dose around the target to be steeply reduced. In a systematic review comparing particle and photon beam therapies for paranasal sinus and nasal cavity tumors, a significantly higher 5‐year DFS rate and local control rate at the longest follow‐up were reported with proton therapy than with IMRT for photon beams.^12^ However, the frequency of neurotoxicity was significantly higher in the particle therapy group than in the photon therapy group, and there were no differences in other adverse events between the two groups (*p* = 0.0002). This could be biased because patients who were anatomically difficult to treat were referred to particle therapy facilities. However, the incidence of Grade 3–4 late adverse events (such as visual impairment and central nervous system necrosis) was not exactly low for particle therapy of nasal sinus tumors (14%–24%) in other studies,^13^, ^14^ indicating that the nasal sinuses are adjacent to anatomically significant organs. Moreover, reduction of the radiation dose to normal tissue is difficult, even with particle beams.

Recently, the potential benefits of preoperative radiotherapy have been reported. A retrospective study showed significantly higher rates of complete resection with negative margins and OS in patients treated with preoperative radiotherapy than in those treated with postoperative radiotherapy or radiotherapy alone.^27^ The optimal timing of radiotherapy should be investigated in future prospective studies. However, there are no clear recommendations regarding the irradiation field in radiation therapy. In our hospital, the irradiation field is defined as the local area, including the surgical bed, and the maxillary sinus on the affected side. In some cases, the maxillary sinus on the affected side was not included in the irradiation field if the continuity of the medial wall of the maxillary sinus remained clear on CT. However, one patient experienced recurrence within the maxillary sinus on the affected side at the edge of the irradiated area after 37 months of follow‐up. Considering the possibility of residual tumor cells in the maxillary sinus that are also washed off at the end of surgery and that the inclusion of the maxillary sinus in the irradiation fields does not increase the incidence of serious adverse events, we believe that the maxillary sinus on the affected side should be included in the local irradiation field.

However, the role of prophylactic cervical lymph node irradiation in radiotherapy for ONB remains controversial. Although ONB is typically localized, cervical lymph node metastasis occurs in approximately 15%–30% of cases.^10^ In some cases, it occurs 5 years after surgery and has been reported as a poor prognostic factor.^2^, ^10^, ^11^, ^16^ Although some retrospective studies have suggested that only local irradiation and salvage surgery can be performed in case of recurrence,^20^ many recent reports recommend prophylactic lymph node irradiation.^2^, ^11^, ^19^ In a retrospective study of 139 cases by Abdelmeguid et al., only 2 (6.4%) of 31 patients who underwent prophylactic cervical lymph node irradiation had cervical recurrence, and both cases had lymph node recurrence outside the irradiation field.^2^ However, as the tumor is located in the nasal cavity, which lies in the midline of the body, lymph node metastasis to the unaffected side is also common. When prophylactic cervical lymph node irradiation is performed, the recommended irradiation fields are bilateral levels Ib, II, and retropharyngeal nodes,^19^ which raise concerns regarding the side effects directly related to a decrease in quality of life, such as oral mucositis and salivation disorders. We do not perform prophylactic cervical lymph node irradiation because many patients have a feeling of rejection to radiation exposure due to the historical background in Japan, and they can visit a hospital regularly after treatment because of the Japanese universal health insurance system. We are in an environment where we can perform cervical neck dissection within 1 month if there is a suspicion of lymph node recurrence. Six of the nine recurrent cases at our hospital showed only cervical lymph node recurrence, and the most common site was Level II on the affected side. However, there was also recurrence at Levels Ib and III on the unaffected side (Figure 4). It has also been reported that 60% of patients with lymph node metastases develop distant metastases.^27^ In our hospital, three of the six patients who had only cervical lymph node metastasis at the time of diagnosis of recurrence were treated with neck dissection with or without postoperative radiotherapy as salvage treatment, and complete response (CR) was maintained; however, the other three patients subsequently developed distant metastases. All three cases with distant metastasis after salvage treatment were Kadish stage C, two were Hyams Grade III, and two had positive margins. Prophylactic lymph node irradiation might be considered only in cases of Kadish stage C disease with other risks of recurrence, such as advanced Hyams grade or positive margins, or in cases where regular long‐term follow‐up is difficult. Therefore, a randomized controlled trial is needed to select patients who require prophylactic lymph node irradiation. Due to the rarity of this disease, it is difficult to collect cases from a single institution; therefore, future multicenter studies are warranted.

We acknowledge that our study has some limitations. Due to our study's retrospective nature and relatively small sample size in a single institution, it is difficult to draw a definitive conclusion on the optimal irradiation fields or the best management for ONB. Furthermore, due to the lack of evidence for the benefits of combining chemotherapy with radiotherapy, we did not administer chemotherapy in all cases. However, it has recently been reported that induction chemotherapy, especially for high‐grade tumors, improves local and regional control rates.^28^ Notwithstanding the above limitations, we believe that this study can show the importance of cooperation between radiation oncologists, head and neck surgeons, and pathologists for good local control.

## 5 CONCLUSION

Patients with ONB treated with postoperative radiation therapy at our hospital had a good local control rate of 100% and a 3‐year OS rate of 95.5%. There were no cases of local recurrence. This may be because the head and neck surgeons removed the tumors using en bloc rather than piecemeal resection, and the pathologists made pathological specimens in the coronal section, which enabled accurate matching of the planning CT images with pathological results, such as the location of the tumor and positive margin, at the time of radiation therapy planning.

Although we did not perform prophylactic cervical lymph node irradiation, 6 (66.7%) of the 9 recurrent cases showed cervical lymph node recurrence. Three of the six patients who had only cervical lymph node metastasis at the time of diagnosis of recurrence were treated with neck dissection with or without postoperative radiotherapy as salvage treatment, and CR was maintained; however, the other three patients subsequently developed distant metastases. All three cases with distant metastasis after salvage treatment were of Kadish Stage C, two were of Hyams Grade III, and two had positive margins. Prophylactic lymph node irradiation might be considered only in cases of Kadish Stage C disease with other risks of recurrence, such as advanced Hyams grade or positive margins, or in cases where regular long‐term follow‐up is difficult.

However, ONB often recurs more than 5 years after the initial definitive treatment, and further long‐term follow‐up is desirable to prove the effectiveness of our multidisciplinary approach and postoperative radiotherapy only for the local area with strict follow‐up.

## AUTHOR CONTRIBUTIONS

**Yuki Tsutsumi:** Conceptualization (equal); data curation (equal); formal analysis (lead); investigation (lead); methodology (equal); project administration (lead); resources (equal); supervision (lead); validation (lead); visualization (lead); writing – original draft (equal); writing – review and editing (lead). **Kazuhiro Omura:** Conceptualization (equal); data curation (equal); methodology (equal); resources (equal); writing – original draft (equal). **Yoshikazu Kijima:** Data curation (supporting); writing – original draft (supporting). **Masao Kobayashi:** Data curation (supporting); writing – original draft (supporting). **Nei Fukasawa:** Methodology (supporting); resources (supporting). **Teppei Takeda:** Resources (supporting). **Teru Ebihara:** Resources (supporting). **Satoshi Aoki:** Resources (supporting). **Nobuyoshi Otori:** Project administration (supporting). **Hiromi Kojima:** Project administration (supporting). **Manabu Aoki:** Conceptualization (supporting); project administration (supporting); resources (supporting); writing – original draft (supporting).

## CONFLICT OF INTEREST STATEMENT

The authors have no conflicts of interest to declare.

## Data Availability

The data were generated at Jikei University School of Medicine. Derived data supporting the findings of this study are available from the corresponding author Y.T on request.
